# Alterations in the Neuromuscular Control Mechanism of the Legs During a Post-Fatigue Landing Make the Lower Limbs More Susceptible to Injury

**DOI:** 10.3390/bioengineering12030233

**Published:** 2025-02-24

**Authors:** Penglei Fan, Youngsuk Kim, Dong-Wook Han, Sukwon Kim, Ting Wang

**Affiliations:** 1 College of Education and Sports Sciences, Yangtze University, Jingzhou 434020, China; fanpenglei@jbnu.ac.kr; 2 Department of Physical Education, Jeonbuk National University, Jeonju 54896, Republic of Korea; ys43530@jbnu.ac.kr; 3 Department of Sport Science, Jeonbuk National University, Jeonju 54896, Republic of Korea; handw@jbnu.ac.kr

**Keywords:** muscle synergy, basketball player, EMG, non-negative matrix factorization

## Abstract

Fatigue causes the lower limb to land in an injury-prone state, but the underlying neuromuscular control changes remain unclear. This study aims to investigate lower limb muscle synergies during landing in basketball players, both before and after fatigue, to examine alterations in neuromuscular control strategies induced by fatigue. Eighteen male recreational basketball players performed landing tasks pre- and post-fatigue induced by 10 × 10 countermovement jumps. Electromyographic (EMG) data from eight muscles, including the erector spinae (ES), rectus abdominus (RA), gluteus maximus (GM), rectus femoris (RF), biceps femoris (BF), lateral gastrocnemius (LG), soleus (SM), and tibialis anterior (TA) muscles, were analyzed using non-negative matrix factorization to extract muscle synergies. Post-fatigue results revealed significant changes: synergy primitive 1 decreased before landing (18–30% phase) and synergy primitive 2 decreased after landing (60–100% phase). Muscle weights of the LG and SM in synergy module 2 increased. Fatigue reduced synergistic muscle activation levels, compromising joint stability and increasing knee joint loading due to greater reliance on calf muscles. These changes heighten the risk of lower limb injuries. To mitigate fatigue-induced injury risks, athletes should improve thigh muscle endurance and enhance neuromuscular control, fostering better synergy between thigh and calf muscles during fatigued conditions.

## 1. Introduction

Landing following a jump requires prevalent lower limb movement and is a common movement for causing injuries in various sports [1,2]. Fatigue promotes the development of high-risk joint neuro-mechanical strategies during landing [3,4,5,6]. This may be related to decreases in muscle strength, proprioception sensitivity, joint laxity, and the ability of muscle fibers to absorb energy [7,8]. These decreases result in decreased hip flexion, increased knee flexion, increased valgus angles and moments, increased vertical ground reaction force (GRF), effects on neuromuscular control, and decreased body stability during landing [3,4,5,9], all of which are clear manifestations of an injury-prone state.

Basketball, compared to other sports, exhibits a higher susceptibility to landing injuries due to the frequent occurrences of rapid body acceleration and deceleration during rebounding, passing, shooting, dunking, and stealing [1,10]. To meet these athletic demands, athletes employ stretch-shortening cycles (SSC) to enhance strength and power [10,11]. However, extensive SSC activity can lead to muscular fatigue, causing a short-term decrease in strength and jumping ability, accompanied by an increase or decrease in muscle-tendon stiffness, eventually altering landing mechanics [11]. Nevertheless, the precise changes during landing in intrinsic neuromuscular control strategies of the lower limb following muscular fatigue remain unclear.

Neuromuscular control strategies can be evaluated by muscle synergy [12]. In the composition of motor execution within the human central nervous system (CNS), the CNS coordinates the activation of multiple muscles in a spatially and temporally synchronized manner. It is referred to as muscle synergy, which is a module consisting of multiple muscles interacting in a weighted manner and activating simultaneously in time to accomplish a movement [13,14]. Decomposition using surface electromyographic (EMG) signals collected from multiple muscles can be used to extract muscle synergies by representing them as synergy modules (weights assigned to each muscle) and synergy primitives (the degree of activation of synergy modules over time) [13,15].

Fatigue has been shown to affect multi-muscular muscle synergies [16], which are attributed to a neuromuscular adaptive mechanism [17]. A study focusing on fatigue in the gluteus maximus (GM) muscle alone found altered muscle synergies in the lower extremities during landing, suggesting an increased risk of hamstring strain [18]. However, different fatigue regimens may elicit varying effects on landing performance [19]. Considering that fatigue during actual competition and training involves multiple muscles, utilizing SSC-guided fatigue and extracting muscle synergies during dynamic landings might more accurately reflect actual neuromuscular control strategies under fatigue conditions in basketball [11].

This study aims to investigate whether fatigue induces changes in neuromuscular control strategies during landing in basketball players. To explore this, muscle synergies were used to reflect neuromuscular control strategies both pre- and post-fatigue intervention. Any changes observed may be linked to an increased risk of injury. Recognizing these changes could provide critical insights for preventing injuries in athletes. Based on previous research, we hypothesize that fatigue will modify the synergy modules and primitives during landing, thereby altering neuromuscular control strategies and potentially increasing the risk of injury.

## 2. Materials and Methods

### 2.1. Participants

A total of 18 recreational male basketball players were recruited to participate in this experiment (Table 1). Each participant actively participated in basketball games at least three times a week, 2 h per session. Participants had no neuromuscular disorders of the lower limbs, no history of lower limb surgery, and no lower limb injuries within the past six months. Each participant provided written informed consent after a detailed explanation of the testing protocol, the possible risks involved, and their right to terminate participation at any time. All procedures were in accordance with the Declaration of Helsinki 1975, as revised in 1996, and were approved by the Jeonbuk University Ethics Committee (JBNU2022-04-008-002, 1 April 2022).

### 2.2. Experiment Process

**Figure 1:**
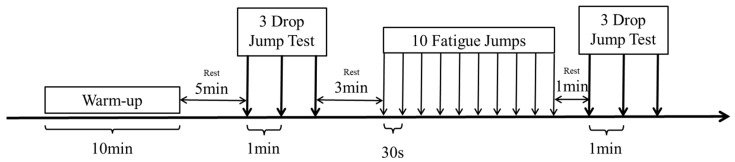
Schematic diagram of the experimental flow.

All experiments were conducted between 15:00 and17:00 each day. Participants were asked to refrain from eating for two hours, consuming caffeine for eight hours, and drinking alcohol for 48 h prior to the experiment. As shown in Figure 1, each participant commenced with a warm-up session comprising five minutes of treadmill running followed by five minutes of dynamic stretching on a wooden floor. Subsequently, a five-minute rest period was observed to allow the body temperature and oxygen consumption to return to baseline levels [20]. The first landing test was then conducted, followed by a three-minute rest period. Fatigue intervention was administered through fatigue jumps, and the second landing test was performed within one minute following the fatigue intervention.

### 2.3. Landing Test

The drop jump test is a commonly used landing test in previous studies to simulate the jump landing activity in basketball for lower limb injury risk assessment [21,22], as it is more consistent with actual sports movements [21]. Participants were instructed to stand on a box 35 cm above the ground with their hands on their chests, without swinging their arms. They were then asked to jump onto a force plate located 35 cm in front of them, land on the force plate, and immediately perform another jump as quickly as possible to reach the highest point. Each participant was required to perform three drop jump tests for each landing, with a one-minute interval between each test, and data were collected for all three landings.

### 2.4. Fatigue Intervention Strategies

To mimic the state of fatigue during actual basketball play, this study employed SSC for fatigue intervention [22]. Each participant completed 10 consecutive sets of 10 countermovement jumps with a 30-s interval between each set, which is a lower extremity fatigue bootstrap that has been used frequently [22,23]. Participants were instructed to stand naturally on the wooden floor with feet shoulder-width apart, place both hands on their waist to minimize arm swing, and perform continuous jumping maneuvers. During the procedure, participants were encouraged and provided with verbal instructions to ensure maximal effort on each jump.

### 2.5. Data Collection

**Figure 2:**
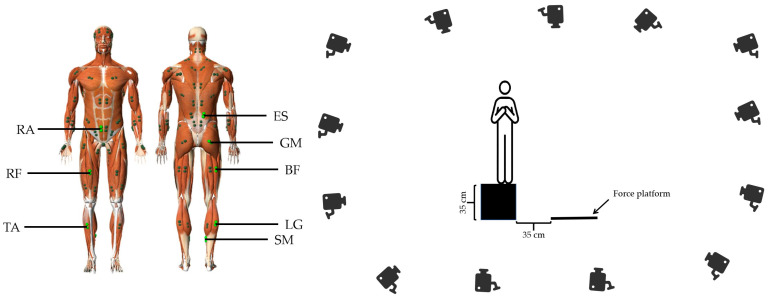
EMG sensor placement position and experimental setup. ES: erector spinae; RA: rectus abdominus; GM: gluteus maximus; RF: rectus femoris; BF: biceps femoris; LG: lateral gastrocnemius; SM: soleus; TA: tibialis anterior.

A 14-mm-diameter reflective marker was used to affix to the participant’s right posterior superior iliac spine (to synchronize the time data), and the trajectory of the marker’s movement was captured at a frequency of 120 HZ using a Motive 2.2.0 (OptiTrack, Natural Point, Inc., Corvallis, OR, USA) motion capture system with 13 infrared cameras. EMG data were collected using eight wireless surface EMG sensors (Trigno Avanti, Delsys, Natick, MA, USA), equipped with dual-differential rod (Ag) electrodes (2.7 × 3.7 cm), at an 8-channel 1200 Hz sampling frequency. The sensors were placed on the dominant side of the lower limb muscles, including the erector spinae (ES), rectus abdominis (RA), gluteus maximus (GM), rectus femoris (RF), biceps femoris (BF), lateral gastrocnemius (LG), soleus (SM), and tibialis anterior (TA) with placement locations referenced to the Atlas of muscle innervation zones (Figure 2) [24]. The sensors were aligned parallel to the muscle fibers [25]. The skin was shaved and swabbed with alcohol swabs prior to placing the sensors [26], and after placing the sensors, surgical waterproof tape (Tegaderm, 3M, St. Paul, MN, USA) was used to secure them [27], ensuring stability during the fatigue intervention, as substantial sweating was expected [28]. GRF data were collected using an OR6-6-2000 force platform (Advanced Mechanical Technology, Inc., Watertown, MA, USA), with a sampling frequency set to 1200 Hz. EMG data and GRF data were captured synchronously in the Motive 2.2.0 motion capture system.

### 2.6. Data Processing

The collected data were imported into Visual 3D software 2024.10.4 (C-Motion, Inc., Germantown, MD, USA) to stage the data. The moment of initial contact was defined when the vertical force was greater than 10 N [29]. EMG data were exported from 100 ms before the moment of initial contact to 100 ms after the moment of initial contact with the ground [30]. This time window was chosen because the degree of muscle pre-activation prior to landing reflects a pre-programmed landing strategy [31]. Additionally, the analysis focused on the first 100 ms after initial ground contact, as this period is critical for understanding landing-related injuries and peak strain loading on the ACL, which typically occurs during this timeframe [32].

**Figure 3:**
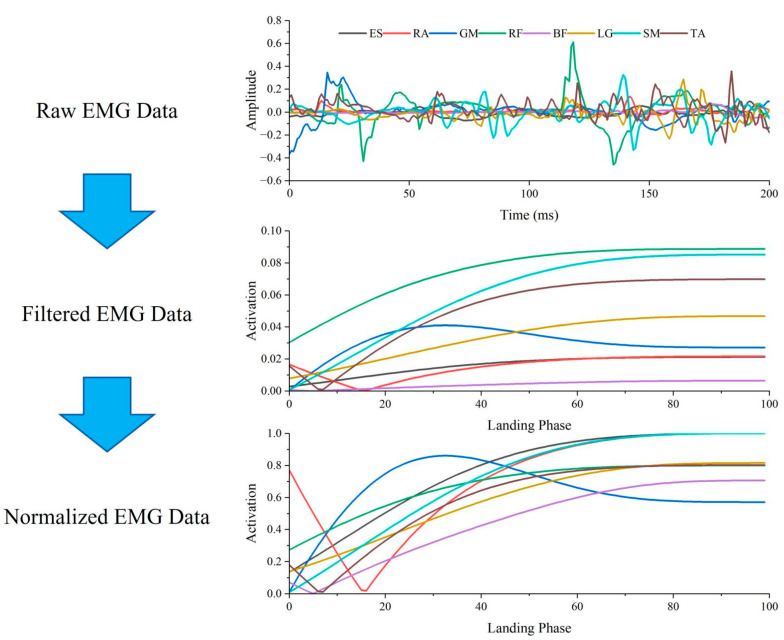
Flowchart of EMG signal processing to obtain the EMG matrix. ES: erector spinae; RA: rectus abdominus; GM: gluteus maximus; RF: rectus femoris; BF: biceps femoris; LG: lateral gastrocnemius; SM: soleus; TA: tibialis anterior.

The processing of the intercepted EMG data of 200 ms at the moment of landing was performed using Matlab R2021a (MathWorks, Natick, MA, USA). The intercepted raw EMG signal was band-pass filtered (20–450 Hz, Butterworth filter, 4th order), full-wave rectified, and smoothed with a zero-lag low-pass filter (6 Hz, Butterworth filter, 4th order) in order to obtain a linear envelope [33]. The signal was then normalized by time to 101 data points (100%), each muscle was normalized to the maximum activity level of the two landing tests, and then the filtered matrix of the raw EMG signal was obtained (Figure 3) [13,15]. In order to show the reproducibility of the EMG data from this experiment, correlation tests were performed on the filtered EMG data from the three measurements in each landing test for each participant. One of the two most correlated datasets (all muscle correlations greater than 0.9) was selected for synergy extraction [34].

In order to decompose the processed EMG matrix and obtain the muscle synergy effect, we used non-negative matrix factorization (NMF) to decompose the original EMG signal matrix with the following algorithmic Formula (1) [35]:

(1) $$EMG\;matrix\;=\;M\;\left(synergy\;modules\right)\;\times \;P\left(synergy\;primitive\right)\;+\;error$$

The EMG matrix is a matrix of raw EMG data processed into m rows and n columns (m = 8 is the number of muscles and n = 101 is the number of normalized time points). M is a matrix of m rows and x columns (x is the number of muscle synergies) representing the muscle weights, that is, synergy modules; P is a matrix of x rows and n columns representing the temporal activation of synergy modules, that is, synergy primitives; and the error is the difference between the initial EMG matrix and the difference between the reconstructed EMG matrix (M × P). The algorithm is based on iteratively updating the initial random guesses of M and P, converging to a locally optimal matrix decomposition. To avoid local minima, it was repeated 20 times per participant. The least-cost solution (i.e., minimizing the squared error between the original and reconstructed EMG patterns) was retained. The EMG matrices pre- and post-fatigue intervention were decomposed separately using this algorithm to obtain the synergy modules and synergy primitives.

To determine the number of synergies, we used the variance accounted for (VAF) (2) for assessment. A VAF of more than 0.95 with a mean VAF increase < 0.01 upon addition of muscle synergy was determined as the number of synergies [36]. It was found that when the number of synergies x = 2, the pre-fatigue intervention VAF = 0.979 ± 0.019 and the post-fatigue intervention VAF = 0.971 ± 0.020, so that both pre-fatigue and post-fatigue intervention muscle synergies could be well explained by two synergies.

(2) $$VAF\;=\;1\;-\;\frac{{\left|EMG\;matrix\;-\;M\;*\;P\;\right|}^{2}}{{\left|EMG\;matrix\;\right|}^{2}}$$

**Figure 4:**
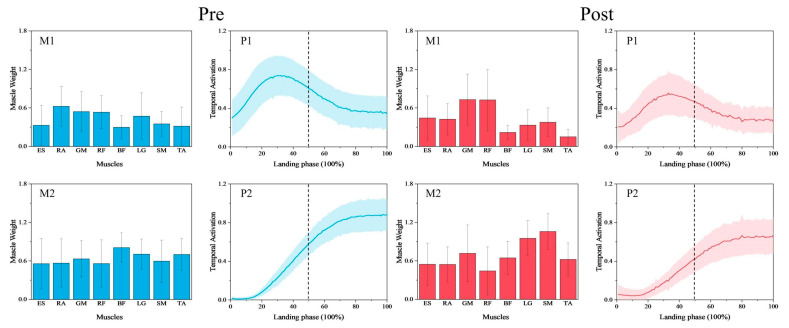
Synergy modules and synergy primitives pre- and post-fatigue intervention in 18 participants (M1 is synergy module 1, M2 is synergy module 2, P1 is synergy primitive 1, and P2 is synergy primitive 2; mean ± SD). In the M-plot, the Y-axis is the weight of the muscle, and the X-axis is the muscle name. In the P-plot, the Y-axis represents the degree of activation of the module, and the X-axis represents the normalized 101 data points, with the 50% dashed line in the middle indicating the moment of initial contact with the ground.

In order to categorize the synergy modules and synergy primitives of the synergies obtained pre- and post-fatigue intervention, we set the number of synergies, x, to a value of k in order to categorize the synergy modules using the k-means clustering algorithm [34,37]. Considering that the k-means solution is affected by the initial center-of-mass clustering prime, it was repeated 50 times for different initial centers of mass to reduce randomness. We also calculated the contour scores of all clustering results to evaluate the optimal clustering results and selected the results with the highest contour scores to output the clustered synergy modules and their corresponding synergy primitives, as shown in Figure 4 [34,37].

### 2.7. Statistical Analysis

To test for the effects of fatigue intervention on synergism, a cosine similarity (CS) test was performed on the synergy modules pre- and post-fatigue intervention [13]. Subsequently, a paired-sample *t*-test was performed on the differences in muscle weights within the synergy modules pre- and post-fatigue intervention [16], with Cohen’s d calculated accordingly as the effect size. A Wilcoxon Signed-Rank test was performed on the weight data for each muscle in all synergistic modules prior to paired-sample *t*-tests. For the test of synergy primitives pre- and post-fatigue intervention, one-dimensional Statistical Parameter Mapping (SPM1d) was used to analyze and compare the activation levels of the modules over the entire time series [37]. The normality assumption of SPM was implicitly checked with the agreement between parametric and non-parametric results [38]. The value of t*, considered equivalent in efficacy to Cohen’s d, was computed to represent the effect size of the test results. All significance levels were set at α < 0.05. Statistical analyses were conducted using Matlab R2021a.

## 3. Results

### 3.1. Comparison of Synergy Modules Pre- and Post-Fatigue Intervention

**Figure 5:**
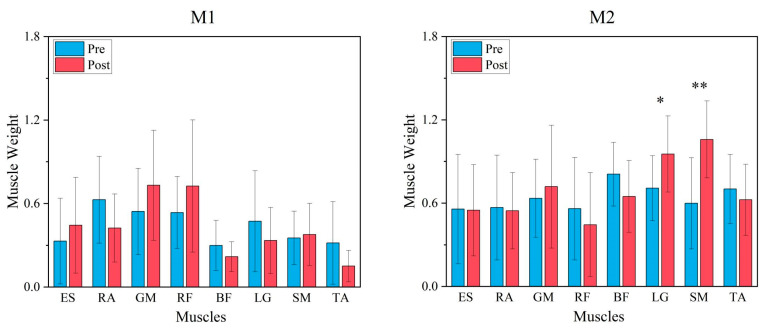
Paired *t*-test results for each muscle in synergy module 1 and synergy module 2 for pre and post-fatigue intervention. Pre-fatigue intervention in blue and post-fatigue intervention in red, * is a significant difference (*p* < 0.05), ** is a very significant difference (*p* < 0.01).

A cosine similarity test of the synergy modules pre- and post-fatigue intervention showed that CS = 0.914 for synergy module 1 and CS = 0.856 for synergy module 2, and both synergy modules were highly similar (CS > 0.8). The paired-samples *t*-test of each of the two synergy modules showed that synergy module 2 showed a significant difference between the muscle weights of the LG (*p* = 0.023, d = −0.965, Figure 5) and a very significant difference between the muscle weights of the SM (*p* < 0.001, d = −1.513, Figure 5) muscles, with no significant difference between the other muscle weights. Whereas in synergy module 1, there was no significant difference in the muscle weights of any muscles.

### 3.2. Comparison of Synergy Primitives Pre- and Post-Fatigue Intervention

**Figure 6:**
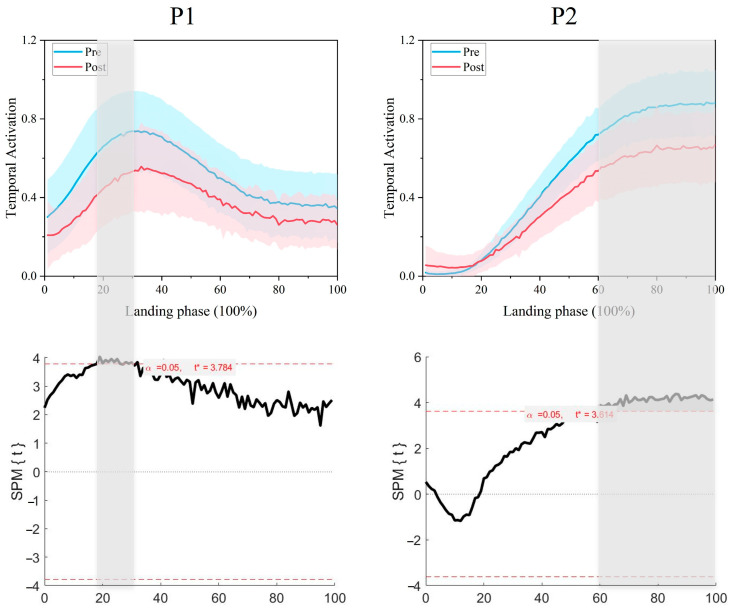
SPM paired samples *t*-test comparing differences between synergy primitives in pre- and post-fatigue interventions. The blue line as pre-fatigue intervention, the red line as post-fatigue intervention, with shading denoting the stage in which the difference occurred.

A paired-sample *t*-test of the synergy primitives SPM1d pre- and post-fatigue intervention showed that synergy primitive 1 showed a significant difference before the initial contact with the ground (18–30% phase, *p* = 0.008, t* = 3.784, Figure 6), and the synergy primitive 2 showed a significant difference after the initial contact with the ground (60–100% phase, *p* < 0.001, t* = 3.614, Figure 6).

## 4. Discussion

Neuromuscular control strategies during lower limb landing, both pre- and post-fatigue, are characterized by two distinct muscle synergies. These two muscle synergies reached their peak values sequentially in the time series: Synergy 1 peaked before ground contact, while Synergy 2 peaked afterward, highlighting distinct phases of neuromuscular control during landing. The limited number of synergies reflects relatively simple neuromuscular control strategies during landing [30,39]. This study also identified significant differences in certain muscle weights within synergy module 2 and specific phases of the time series of both synergy primitives before and after the fatigue condition. Fatigue did not alter the number of synergies but significantly modified their synergistic activation patterns.

Before landing, muscle activation reflects a pre-programmed strategy [31], which is altered by fatigue during landing. Synergy module 1 showed no significant changes in muscle weights, with higher GM and RF weights in the major muscles of hip and knee extension, indicating preserved pre-landing preparatory control under fatigue. Reduced synergy primitive 1 under fatigue may impair preparatory mechanisms, reducing their effectiveness in mitigating impact forces during initial contact. Reduced muscle activation post-fatigue, as linked to the inhibition of spinal and supraspinal centers [22], may disrupt motor control, undermine joint stability, and increase injury susceptibility. This inhibition may decrease the gain of spinal reflex circuitry [40]. Muscle synergy may represent low-dimensional modules stored in the spinal cord or brainstem [14,41], and their fatigue-induced disruption can compromise neuromuscular coordination during landing. Fatigue-induced inhibition of central control centers is likely to reduce the activation of synergy module 1, thereby diminishing the body’s ability to prepare for a safe landing. This reduction might compromise pre-landing joint stiffness, increasing susceptibility to improper load distribution and injury during landing. Fatigue was associated with a decrease in synergy primitive 2 post-contact, potentially reducing the lower limb’s ability to manage impact forces and maintain stability. The reduction in synergy primitive 2 can similarly be attributed to the fatigue-induced inhibition of the spinal cord and supraspinal centers, as observed for synergy primitive 1 [22]. Fatigue has been shown to increase knee flexion, joint laxity, and reduce the ability of muscle fibers to absorb energy [7]. Additionally, Kernozek et al. and Chappell et al. reported that fatigue during landing increases the valgus angle, valgus moment, and anterior knee shear, thereby elevating the risk of ACL injury [4,5]. Research suggests that maintaining knee stability during functional activities requires the surrounding knee muscles to contract simultaneously, or co-activate [42]. A lower degree of co-activation in synergy module 2 may contribute to joint laxity and reduced knee stability. Joint laxity is a potential risk factor for increased valgus collapse of the knee, which imposes higher mechanical loads on the collateral and cruciate ligaments [43], thereby increasing the risk of injury.

Previous studies have shown that fatigue leads to decreased knee moment, increased knee flexion, elevated peak ankle moment, and a greater ankle contribution to the maximum moment during landing [44]. The present study also found an increase in the muscle weights of the calf muscles LG and SM post-fatigue in synergy module 2. Calf muscles are known to contain high levels of slow-twitch fibers [45], especially the LG and SM, have more than 50% slow-twitch fibers [46], which enhance their fatigue resistance during exercise [47]. Other studies have also found an increase in calf muscle activation when using SSC to guide fatigue in the calf muscle and suggest that it is the SSC exercise-induced contractile failure that has an effect on the flexible adjustment of neural activation [48]. Similarly, this study proposes that the increase in calf muscle weights reflects an adjustment in neural flexibility to compensate for reduced synergistic activation of lower limb muscle groups under fatigue. Furthermore, one mechanism of adaptation to fatigue involves greater variability in co-activation between non-fatigued and other muscle groups [17]. The increased weight of the lower leg muscles may, therefore, reflect the nervous system’s attempt to balance the functional decline caused by fatigue by enhancing the coordinated activation of non-fatigued muscle groups. Brazen et al. found that landing after fatigue resulted in greater ankle dorsiflexion angles at initial contact, increased peak GRF, and a longer time required to stabilize the body after landing [3]. The increased weights of the LG and SM muscles suggest an increase in ankle plantar flexor activation, which may lead to greater ankle stiffness [49], enhancing the efficiency of force transmission and potentially contributing to the observed increase in GRF. Research has also shown that repeated impact activities can lead to increased tibial translation [50], and a stiffer ankle joint can transmit more force from the ankle and tibia to the knee. Collectively, these factors may increase the load on the ACL, potentially elevating the risk of injury. Therefore, the increased weight of calf muscles during landing under fatigued conditions may contribute to a state of increased injury risk for the lower limb.

Based on the above findings, fatigue-induced alterations in neuromuscular control strategies during landing may increase the risk of lower limb injuries. Previous studies have shown that fatigue leads to alterations in the muscle synergy patterns of the lower limbs during running [16]. Additionally, isolated GM fatigue can also alter lower limb muscle synergies, increasing the risk of hamstring strains [18]. In this study, we used a more realistic fatigue model, the SSC, to induce fatigue [11] and observed alterations in neuromuscular control strategies and an increased risk of landing injuries under fatigue conditions in basketball. To mitigate the risk of lower limb injuries associated with fatigue-induced changes in neuromuscular control, neuromuscular control training under fatigued conditions, such as Integrative Neuromuscular Training (INT), is recommended. This approach has been shown to effectively reduce the risk of lower limb sports injuries in athletes [51,52]. Studies indicate that such training enhances the central nervous system’s feedforward control mechanisms, thereby improving athletes’ neuromuscular control over postural stability [53]. Additionally, maintaining precise neuromuscular control under fatigue may prevent inadequate lower limb muscle synergistic activation before and after landing. This not only prepares the lower limbs for impact but also maintains joint stability after landing, ultimately enhancing both performance and safety during landing movements. Furthermore, muscle synergy can be improved through targeted training [33]. To address the observed effects of fatigue on neuromuscular control, it is recommended to enhance endurance training for thigh muscles and incorporate dynamic resistance training targeting the coordinated activation of both calf and thigh muscles. This approach may effectively prevent excessive knee or ankle joint loading due to altered lower limb muscle synergistic activation patterns in fatigued states, further reducing the risk of injury associated with fatigue.

This study also has some limitations. Firstly, although it aimed to approximately simulate fatigue during training and competition, it did not incorporate running, changes in direction, or external distractions [54]. While fatigue is correlated with injury occurrence, it is not the sole cause [23]. Factors such as perceptual-cognitive tasks and external distractions may also play a role [55]. Secondly, EMG data were collected from only eight lower limb muscles. Notably, the number of muscle synergies is influenced by the selection and number of muscles analyzed [56]. Additionally, the study lacked female participants, despite evidence that females are three times more likely to experience ACL injuries than males [54,57,58] and may be more susceptible to fatigue-induced changes in neuromuscular control strategies. Therefore, it is recommended that future studies involve a broader population and be conducted in a manner more consistent with actual training and competition conditions.

## 5. Conclusions

Fatigue-induced alterations in neuromuscular control during landing reduce muscle synergistic activation levels both before and after landing. This decline may impair an athlete’s ability to prepare for landing, compromising impact absorption capacity and joint stability. Additionally, increased reliance on calf muscle weights could lead to greater stiffness in the calf muscles, potentially increasing stress on the knee joint. To mitigate injury risks, it is recommended that injury prevention programs incorporate neuromuscular agility training under fatigued conditions. This would help enhance neuromuscular control after fatigue. Furthermore, incorporating endurance training for thigh muscles and exercises to improve coordination between the thigh and calf muscles under fatigue is advised.

## Figures and Tables

**Table 1:** Basic physical characteristics of the 18 participants.

Age (Years)	Height (m)	Weight (kg)	Body Mass Index (kg/m²)	Body Fat Percentage (%)
20.3 ± 1.5	1.81 ± 0.06	76.62 ± 5.24	23.29 ± 2.14	14.3 ± 2.9

## Data Availability

The dataset generated and analyzed during the current study is available from the corresponding author upon reasonable request.

## References

[1] Taylor J.B., Ford K.R., Nguyen A.-D., Terry L.N., Hegedus E.J. (2015). Prevention of lower extremity injuries in basketball: A systematic review and meta-analysis. Sports Health.

[2] Cortes N., Morrison S., Van Lunen B.L., Onate J.A. (2012). Landing technique affects knee loading and position during athletic tasks. J. Sci. Med. Sport.

[3] Brazen D.M.M., Todd M.K., Ambegaonkar J.P.P., Wunderlich R., Peterson C.P. (2010). The effect of fatigue on landing biomechanics in single-leg drop landings. Am. J. Ther..

[4] Kernozek T.W., Torry M.R., Iwasaki M. (2008). Gender Differences in lower extremity landing mechanics caused by neuromuscular fatigue. Am. J. Sports Med..

[5] Chappell J.D., Herman D.C., Knight B.S., Kirkendall D.T., Garrett W.E., Yu B. (2005). Effect of fatigue on knee kinetics and kinematics in stop-jump tasks. Am. J. Sports Med..

[6] Verschueren J., Tassignon B., De Pauw K., Proost M., Teugels A., Van Cutsem J., Roelands B., Verhagen E., Meeusen R. (2020). Does acute fatigue negatively affect intrinsic risk factors of the lower extremity injury risk profile? A systematic and critical review. Sports Med..

[7] Rozzi S.L., Lephart S.M., Fu F.H. (1999). Effects of muscular fatigue on knee joint laxity and neuromuscular characteristics of male and female athletes. J. Athl. Train..

[8] Skinner H.B., Wyatt M.P., Hodgdon J.A., Conard D.W., Barrack R.L. (1986). Effect of fatigue on joint position sense of the knee. J. Orthop. Res..

[9] Heil J., Loffing F., Büsch D. (2020). The Influence of exercise-induced fatigue on inter-limb asymmetries: A systematic review. Sports Med. Open.

[10] Delextrat A., Badiella A., Saavedra V., Matthew D., Schelling X., Torres-Ronda L. (2015). Match activity demands of elite Spanish female basketball players by playing position. Int. J. Perform. Anal. Sport.

[11] Yoshida N., Hornsby W.G., Sole C.J., Sato K., Stone M.H. (2024). Effect of neuromuscular fatigue on the countermovement jump characteristics: Basketball-related high-intensity exercises. J. Strength Cond. Res..

[12] Sawers A., Allen J.L., Ting L.H. (2015). Long-term training modifies the modular structure and organization of walking balance control. J. Neurophysiol..

[13] Santuz A., Ekizos A., Janshen L., Baltzopoulos V., Arampatzis A. (2017). On the methodological implications of extracting muscle synergies from human locomotion. Int. J. Neural Syst..

[14] Bizzi E., Cheung V., D’Avella A., Saltiel P., Tresch M. (2008). Combining modules for movement. Brain Res. Rev..

[15] Safavynia S.A., Torres-Oviedo G., Ting L.H. (2011). Muscle synergies: Implications for clinical evaluation and rehabilitation of movement. Top. Spinal Cord Inj. Rehabil..

[16] Hajiloo B., Anbarian M., Esmaeili H., Mirzapour M. (2020). The effects of fatigue on synergy of selected lower limb muscles during running. J. Biomech..

[17] Singh T., Latash M.L. (2011). Effects of muscle fatigue on multi-muscle synergies. Exp. Brain Res..

[18] Matsunaga N., Okubo Y., Isagawa S., Niitsuma J., Otsudo T., Sawada Y., Akasaka K. (2021). Muscle fatigue in the gluteus maximus changes muscle synergies during single-leg landing. J. Bodyw. Mov. Ther..

[19] James C.R., Scheuermann B.W., Smith M.P. (2010). Effects of two neuromuscular fatigue protocols on landing performance. J. Electromyogr. Kinesiol..

[20] Li M., Meng X., Guan L., Kim Y., Kim S. (2023). Comparing the effects of static stretching alone and in combination with post-activation performance enhancement on squat jump performance at different knee starting angles. J. Sports Sci. Med..

[21] Taylor K., Terry M., Utturkar G., Spritzer C., Queen R., Irribarra L., Garrett W., DeFrate L. (2011). Measurement of in vivo anterior cruciate ligament strain during dynamic jump landing. J. Biomech..

[22] Lazaridis S., Patikas D.A., Bassa E., Tsatalas T., Hatzikotoulas K., Ftikas C., Kotzamanidis C. (2018). The acute effects of an intense stretch-shortening cycle fatigue protocol on the neuromechanical parameters of lower limbs in men and prepubescent boys. J. Sports Sci..

[23] Madigan M.L., Pidcoe P.E. (2003). Changes in landing biomechanics during a fatiguing landing activity. J. Electromyogr. Kinesiol..

[24] Barbero M., Merletti R., Rainoldi A. (2012). Atlas of Muscle Innervation Zones: Understanding Surface Electromyography and Its Applications.

[25] Yoon S., Bailey C.A., Cohen N.R., Côté J.N. (2021). Changes in muscle activation, oxygenation, and morphology following a fatiguing repetitive forward reaching task in young adult males and females. J. Electromyogr. Kinesiol..

[26] Merletti R., Cerone G. (2020). Tutorial. Surface EMG detection, conditioning and pre-processing: Best practices. J. Electromyogr. Kinesiol..

[27] Martire R.L., Gladh K., Westman A., Äng B.O. (2017). Neck muscle Emg-force relationship and its reliability during isometric contractions. Sports Med. Open.

[28] Castillo-Lozano R., Cuesta-Vargas A.I. (2013). A comparison land-water environment of maximal voluntary isometric contraction during manual muscle testing through surface electromyography. J. Sports Med. Arthrosc. Rehabil. Ther. Technol..

[29] Gholipour Aghdam G.M., Alizadeh M.H., Minoonejad H., Shirzad E., Wilke J. (2024). Knee biomechanics during neurocognitively challenged drop landings in male elite soccer players with anterior cruciate ligament reconstruction. Sports Med. Open.

[30] Kipp K., Pfeiffer R., Sabick M., Harris C., Sutter J., Kuhlman S., Shea K. (2014). muscle synergies during a single-leg drop-landing in boys and girls. J. Appl. Biomech..

[31] Horita T., Komi P., Nicol C., Kyröläinen H. (2002). Interaction between pre-landing activities and stiffness regulation of the knee joint musculoskeletal system in the drop jump: Implications to performance. Eur. J. Appl. Physiol..

[32] Koga H., Muneta T., Muneta T., Sekiya I. (2016). ACL injury mechanisms. ACL Injury and Its Treatment.

[33] Fan P., Yang Z., Wang T., Li J., Kim Y., Kim S. (2024). Neuromuscular control strategies in basketball shooting: Distance-dependent analysis of muscle synergies. J. Sports Sci. Med..

[34] Aoyama T., Ae K., Kohno Y. (2022). Interindividual differences in upper limb muscle synergies during baseball throwing motion in male college baseball players. J. Biomech..

[35] Lee D., Seung H.S., Dietterich T.G., Becker S., Ghahramani Z. (2000). Algorithms for non-negative matrix factorization. Advances in Neural Information Processing Systems.

[36] Li Z., Zhao X., Wang Z., Xu R., Meng L., Ming D. (2022). A hierarchical classification of gestures under two force levels based on muscle synergy. Biomed. Signal Process. Control.

[37] Pan Z., Liu L., Li X., Ma Y. (2024). Characteristics of muscle synergy and anticipatory synergy adjustments strategy when cutting in different angles. Gait Posture.

[38] Baumgart C., Kurz E., Freiwald J., Hoppe M.W. (2021). Effects of hip flexion on knee extension and flexion isokinetic angle-specific torques and Hq-ratios. Sports Med. Open.

[39] Stoloff R.H., Zehr E.P., Ferris D.P. (2006). Recumbent stepping has similar but simpler neural control compared to walking. Exp. Brain Res..

[40] Ricotta J.M., De S.D., Nardon M., Benamati A., Latash M.L. (2023). Effects of fatigue on intramuscle force-stabilizing synergies. J. Appl. Physiol..

[41] Latash M.L. (2010). Motor Synergies and the equilibrium-point hypothesis. Mot. Control.

[42] Akl A.-R., Conceição F., Richards J. (2023). An exploration of muscle co-activation during different walking speeds and the association with lower limb joint stiffness. J. Biomech..

[43] Shultz S.J., Schmitz R.J. (2009). Effects of transverse and frontal plane knee laxity on hip and knee neuromechanics during drop landings. Am. J. Sports Med..

[44] Orishimo K.F., Kremenic I.J. (2006). Effect of fatigue on single-leg hop landing biomechanics. J. Appl. Biomech..

[45] Gaffney C.J., Fomina E., Babich D., Kitov V., Uskov K., Green D.A. (2017). The effect of long-term confinement and the efficacy of exercise countermeasures on muscle strength during a simulated mission to Mars: Data from the Mars500 study. Sports Med. Open.

[46] Edgerton V.R., Smith J.L., Simpson D.R. (1975). Muscle fibre type populations of human leg muscles. Histochem. J..

[47] Place N., Yamada T., Bruton J.D., Westerblad H. (2010). Muscle fatigue: From observations in humans to underlying mechanisms studied in intact single muscle fibres. Eur. J. Appl. Physiol..

[48] Regueme S.C., Nicol C., Barthèlemy J., Grélot L. (2005). Acute and delayed neuromuscular adjustments of the triceps surae muscle group to exhaustive stretch? shortening cycle fatigue. Eur. J. Appl. Physiol..

[49] Riemann B.L., DeMont R.G., Ryu K., Lephart S.M. (2001). The effects of sex, joint angle, and the gastrocnemius muscle on passive ankle joint complex stiffness. J. Athl. Train..

[50] Kvist J., Cunningham D., Tigerstrand-Wejlemark H. (2006). Gender differences in post-exercise sagittal knee translation: A comparison between elite volleyball players and swimmers. Knee.

[51] Zhai H., Li C., Xia J., Wei H., Qin S. (2022). Integrative neuromuscular training for injury prevention of lower extremity in athletes: A meta-analysis. Chin. J. Tissue Eng. Res..

[52] Belamjahad A., Tourny C., Jebabli N., Clark C.C.T., Laher I., Hackney A.C., Granacher U., Zouhal H. (2024). Effects of a preseason neuromuscular training program vs. an endurance-dominated program on physical fitness and injury prevention in female soccer players. Sports Med. Open.

[53] Zemková E., Zapletalová L. (2022). The role of neuromuscular control of postural and core stability in functional movement and athlete performance. Front. Physiol..

[54] Donelon T.A., Edwards J., Brown M., Jones P.A., O’driscoll J., Dos’santos T. (2024). Differences in biomechanical determinants of Acl injury risk in change of direction tasks between males and females: A systematic review and meta-analysis. Sports Med. Open.

[55] Mejane J., Faubert J., Romeas T., Labbe D.R. (2019). The combined impact of a perceptual–cognitive task and neuromuscular fatigue on knee biomechanics during landing. Knee.

[56] Steele K.M., Tresch M.C., Perreault E.J. (2013). The number and choice of muscles impact the results of muscle synergy analyses. Front. Comput. Neurosci..

[57] Prodromos C.C., Han Y., Rogowski J., Joyce B., Shi K. (2007). A Meta-analysis of the incidence of anterior cruciate ligament tears as a function of gender, sport, and a knee injury–reduction regimen. Arthrosc. J. Arthrosc. Relat. Surg..

[58] Yoshida N., Kunugi S., Mashimo S., Okuma Y., Masunari A., Miyazaki S., Hisajima T., Miyakawa S. (2016). Effect of forefoot strike on lower extremity muscle activity and knee joint angle during cutting in female team handball players. Sports Med. Open.

